# Integrated network toxicology, machine learning algorithms and TMT proteomics reveal the mechanism of 18β glycyrrhetinic acid against gastric cancer

**DOI:** 10.3389/fgene.2025.1688077

**Published:** 2026-01-06

**Authors:** Doudou Lu, Shumin Jia, Yahong Li, Zhaozhao Wang, Ziying Zhou, Wenjing Liu, Lei Zhang, Ling Yuan, Yi Nan

**Affiliations:** 1 School of Basic Medicine, Ningxia Medical University, Yinchuan, Ningxia, China; 2 Traditional Chinese Medicine College, Ningxia Medical University, Yinchuan, Ningxia, China; 3 Department of Pharmacy, General Hospital of Ningxia Medical University, Yinchuan, Ningxia, China; 4 Key Laboratory of Hui Ethnic Medicine Modernization of Ministry of Education, Ningxia Medical University, Yinchuan, Ningxia, China; 5 College of Pharmacy, Ningxia Medical University, Yinchuan, Ningxia, China

**Keywords:** 18β glycyrrhetinic acid, TMT proteomics, machine learning algorithms, NEDD4L, gastric cancer

## Abstract

The purpose of this paper is to explore the mechanism of 18β glycyrrhetinic acid (18β-GRA) in treating gastric cancer. Firstly, the toxicological effects of 18β-GRA were predicted using the ProTox3.0 database. Then, candidate biomarkers for the anti-gastric cancer of 18β-GRA were screened using the weighted gene co-expression network analysis (WGCNA), the least absolute shrinkage and selection operator (LASSO), the support vector machine (SVM), the random forest algorithm combined with the TMT proteomics methods. Additionally, we explored the potential upstream transcription factors and downstream interacting proteins of the biomarkers. The WGCNA method yielded 269 targets, while TMT proteomics analysis identified 6,273 genes. Among these, 12 targets were identical. Using LASSO, SVM, and random forest algorithms, three candidate markers were identified: insulin-like growth factor 2 mRNA binding protein 3 (IGF2BP3), keratin 6B (KRT6B), and E3 ubiquitin-protein ligase NEDD4-like (NEDD4L). Based on molecular docking and molecular dynamics results, NEDD4L is believed to be a 18β-GRA biomarker, while sodium channel protein type 5 subunit alpha (SCN5A) and early growth response protein 1 (EGR1) are the potential upstream and downstream regulatory proteins, respectively. These findings provide a theoretical basis for future experimental verification.

## Graphical Abstract

**Figure:**
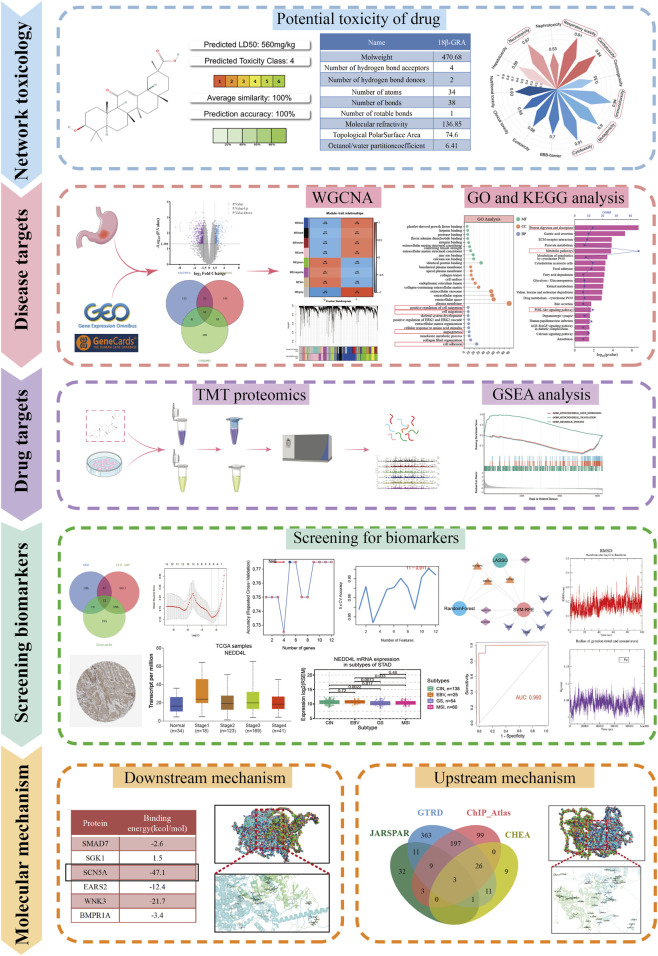

## 1 Introduction

Gastric cancer is one of the most prevalent malignancies worldwide, characterized by high morbidity and mortality rates. According to the 2020 report, approximately 1,089,103 new cases of gastric cancer were diagnosed, resulting in 768,793 deaths annually (Sung et al., 2021). Due to the lack of specific and sensitive biomarkers, most patients are not eligible for surgical resection by the time they are diagnosed. Current treatment options include surgical resection, radiation therapy, chemotherapy, and immunosuppressive drugs. However, the overall 5-year survival rate remains below 50% (Li et al., 2022). Therefore, understanding the molecular mechanisms underlying gastric cancer progression is crucial for improving diagnosis and treatment. Molecular targeted therapy uses drugs to block specific molecules in cancer cells, enhancing treatment specificity and reducing damage to normal cells. Agents such as trastuzumab and ramucirumab have shown greater effectiveness when combined with chemotherapy (Bang et al., 2010; Fuchs et al., 2014; Wilke et al., 2014; Shitara et al., 2020). Consequently, identifying novel molecular biomarkers holds significant promise for developing new gastric cancer therapies.

Due to the unique structures, biocompatibility, and low toxicity, natural small molecules are considered to be more promising therapeutic agents than biological macromolecules (Hou et al., 2022). Licorice, one of the most widely used herbs in traditional Chinese medicine, contains several active ingredients, including glycyrrhizic acid, liquiritin, and others (Wang et al., 2021). Upon ingestion, glycyrrhizic acid rapidly metabolizes into its aglycones: 18α glycyrrhetinic acid (18α-GRA) and 18β glycyrrhetinic acid (18β-GRA) (Li et al., 2019). The distinct spatial orientation of the hydrogen atom at C18 (C18-H) in these stereoisomers results in different biological activities. Studies have confirmed that the β configuration exhibits stronger target binding affinity and greater stability than the α configuration (Sun et al., 2018), and plays significant roles in anti-inflammatory, antiviral, immunoregulatory, and organ-protective activities (Chen et al., 2021; Yi et al., 2022; Wei et al., 2024; Cheng Y. et al., 2022; Jiang et al., 2024). Our previous research demonstrated in cellular and animal models that 18β-GRA significantly inhibits gastric cancer cell survival, induces apoptosis, arrests the cell cycle, and suppresses clonogenic ability. Furthermore, 18β-GRA inhibits the growth of subcutaneous tumors in xenograft mouse models (Yuan et al., 2022; Li et al., 2023; Yang et al., 2023). However, the specific molecular mechanism of 18β-GRA in anti-cancer effect remains unclear.

Advances in computing technology have made it possible to acquire large-scale tumor sample data from public databases, thereby enhancing the realism and comprehensiveness of research outcomes. In this study, we screened for potential 18β-GRA biomarkers using tandem mass tag (TMT) proteomics coupled with machine learning approaches, including the weighted gene co-expression network analysis (WGCNA), the least absolute shrinkage and selection operator (LASSO), the support vector machines (SVMs), and the random forest method. Public databases were also used to predict the regulatory mechanisms of these candidate targets, providing a theoretical basis for subsequent experimental validation. Beyond investigating biological activity, assessing drug safety is critical. We applied network toxicology, an integrative approach combining bioinformatics, systems biology, and toxicology, to evaluate the potential organ toxicity, toxicological endpoints, and associated toxic pathways of 18β-GRA (Banerjee et al., 2024). This provides a novel research model for practical applications. The overall study design is summarized in the Graphical abstract.

## 2 Materials and methods

### 2.1 Potential toxic effects of 18β-GRA

Compound toxicity profiles were predicted using the ProTox3.0 database (https://tox.charite.de/protox3/). This tool evaluates organ-specific toxicity, molecular initiating events, Tox21 adverse outcome pathways, and provides categorical toxicity predictions with associated confidence scores. ProTox 3.0 database was used to predict the acute oral toxicity (LD50 and toxicity class) of 18β-GRA, as well as organ toxicity (hepatotoxicity, neurotoxicity, nephrotoxicity, respiratory toxicity and cardiotoxicity), toxicity end points (carcinogenicity, immunotoxicity, mutagenicity, cytotoxicity, BBB-barrier, ecotoxicity, clinical toxicity and nutritional toxicity), and toxicological pathways (Tox21 nuclear receptor signaling pathways, Tox21 stress response pathways, Molecular initiating events and Metabolism). Following retrieval of the standardized compound name from the PubChem database (https://pubchem.ncbi.nlm.nih.gov/), “18β glycyrrhetinic acid” was submitted to ProTox3.0 for analysis. The results were visualized as radar charts and bar graphs.

### 2.2 Screening for gastric cancer targets

Potential gastric cancer targets were identified by screening the gene expression omnibus (GEO) database (https://www.ncbi.nlm.nih.gov/geo/). We selected datasets derived from *Homo sapiens* that contained both gastric cancer tissues and adjacent non-cancerous tissues. Differentially expressed genes (DEGs) were defined as genes satisfying |logFC| ≥ 1 with an adjusted *p-value* < 0.05. Genes with logFC ≤ −1 were considered downregulated, while those with logFC ≥1 were considered upregulated. The intersection of DEGs across the selected datasets was determined using the Venny tool (https://bioinfogp.cnb.csic.es/tools/venny/). All gene names were standardized to official nomenclature using the UniProt database (https://www.uniprot.org/).

The WGCNA was used to construct co-expression networks and analyze the dataset retrieved from the GEO database. First, genes with low expression variance were eliminated. Next, a weighted gene co-expression network was constructed for all remaining genes using the WGCNA package in R, and the adjacency matrix was then transformed into a topological overlap matrix (TOM) to estimate the degree of shared neighbors between gene pairs. Hierarchical clustering based on the TOM dissimilarity was performed to generate a cluster dendrogram. Genes were dynamically assigned to modules using a dynamic tree-cutting algorithm. The module eigengenes representing the first principal component of each module were then calculated. Genes demonstrating the highest module membership within the key modules were identified as hub genes and selected for further functional investigation.

### 2.3 Functional enrichment analysis of gastric cancer targets

Candidate gastric cancer targets were identified by intersecting DEGs with key genes derived from the WGCNA method. Functional enrichment analysis was performed on candidate targets to elucidate biological processes (BP), molecular functions (MF), cellular components (CC), and signaling pathways. This analysis utilized the database for annotation, visualization, and integrated discovery (DAVID) (https://david.ncifcrf.gov/) for gene ontology (GO) terms and kyoto encyclopedia of genes and genomes (KEGG) pathways. Significantly enriched terms and pathways (*p-value* < 0.05) were identified for further analysis.

### 2.4 TMT proteomics

TMT-based quantitative proteomics uses isobaric labeling at the peptide level coupled with tandem mass spectrometry (MS/MS) to determine relative protein abundance. Since the proteomic experiments of 18β-GRA have been described in our previous studies, the experimental procedure is briefly described here (Yang et al., 2023). Briefly, the AGS gastric cancer cells were treated with 18β-GRA (purity >97%, G10105-10G, Sigma, USA) at a concentration of 78 μmol/L for 24 h. AGS gastric cancer cells were treated with 18β-GRA for 24 h. Following treatment, the cells were harvested and lysed. Total protein concentration was quantified using a bicinchoninic acid (BCA) assay, and then the protein extracts were then reduced with dithiothreitol (DTT), alkylated with iodoacetamide (IAA), and digested with trypsin. The resulting peptides were desalted and labeled with TMT reagents according to the manufacturer’s instructions (Thermo scientific TMT labeling kit). The combined peptide mixture was fractionated using high-pH reversed-phase chromatography on an Agilent 1260 Infinity II HPLC system. The mobile phases were buffer A (a 0.1% aqueous formic acid solution) and buffer B (an 80% acetonitrile solution containing 0.1% formic acid). The fractionated peptides were analyzed using nano-liquid chromatography coupled online with a Q Exactive Plus hybrid quadrupole-orbitrap mass spectrometer to identify and quantify the peptides. To ensure statistical reliability, this experiment was conducted with three independent biological replicates.

### 2.5 Identification of 18β-GRA therapeutic biomarkers in gastric cancer

To address the potential limitations in target coverage from the GEO database alone, we integrated gastric cancer-associated targets from the GeneCards database (https://www.genecards.org/). Common targets intersecting the GEO, GeneCards, and TMT proteomics datasets were identified. The differential expression of these targets was visualized using bar plots. The LASSO, SVMs, and random forests were used for biomarker screening. The LASSO is a penalized linear regression model that selects features by shrinking non-informative coefficients to zero. This improves prediction accuracy and identifies key variables. The SVMs is a kernel-based classifier that constructs optimal hyperplanes to maximize the margin between classes, which allows it to effectively handle high-dimensional feature spaces. The random forest is an ensemble method that aggregates predictions from multiple decision trees trained on bootstrapped samples, with final classification determined by majority voting. These algorithms were implemented in R using the glmnet (LASSO), e1071 (SVM), and randomForest packages. We obtained the potential biomarkers for gastric cancer treatment from 18β-GRA by intersecting the results of the three machine learning methods.

### 2.6 Molecular docking

The molecular docking methodology for compound-protein interactions follows our previously published protocol (Lu et al., 2024). This section describes the procedure for protein-protein docking. First, we retrieved the protein structure in PDB format from the alphaFold protein structure database (https://alphafold.com/). Then, the structure was submitted to the GRAMM tool (https://gramm.compbio.ku.edu/) for docking. Next, we used the PDBePISA database (https://www.ebi.ac.uk/pdbe/pisa/) to analyze the resulting complexes and characterize the interaction interface, amino acids, interaction regions, surface area proportions, hydrogen bonds, and binding affinity. Finally, the complex exhibiting the lowest binding energy was visualized using PyMOL software.

### 2.7 Molecular dynamics simulation

Molecular dynamics (MD) simulations were performed using GROMACS 2021.5. The simulation procedure consisted of the following steps: First, the 18β-GRA compound was parameterized with the AMBER force field using the Acpype server (https://bio2byte.be/acpype/). Then, the NEDD4L protein was parameterized with the AMBER force field. The 18β-GRA-NEDD4L complex was solvated in a cubic box filled with TIP3P water molecules, with a minimum distance of 1.0 nm ensured between the complex and the box boundary. An appropriate number of Cl^−^ counterions were added to neutralize the system charge. Then, energy minimization was conducted using the steepest descent algorithm to remove steric clashes. The system then underwent equilibration with 100 ps of NVT ensemble simulation, followed by 100 ps of NPT ensemble simulation. Finally, a 100-ns production simulation was performed under NPT conditions at 300 K and 1 bar. Trajectory analysis included the following: Trajectory analysis included: root mean square deviation (RMSD) of the protein backbone using gmx rms, root mean square fluctuation (RMSF) of C-α atoms using gmx rmsf, radius of gyration (Rg) using gmx gyrate, solvent accessible surface area (SASA) using gmx sasa, and ligand-protein hydrogen bond analysis using gmx hbond. All dynamic data were visualized using QTgrace 5.1.25.

### 2.8 Clinical correlation and immune infiltration analysis

To further explore the clinical value of candidate biomarkers, we investigated relevance using multiple databases. The mRNA expression levels were analyzed using the university of alabama at birmingham cancer data analysis portal (UALCAN) database (https://ualcan.path.uab.edu/). The cellular localization of targets was determined in the human protein atlas (HPA) database (https://www.proteinatlas.org/). Additionally, the relationship between target expression and gastric cancer molecular subtypes was assessed using the gene set cancer analysis (GSCA) database (https://guolab.wchscu.cn/). The correlations between target expression and survival prognosis were evaluated by kaplan-meier plotter database (https://kmplot.com/). Finally, the receiver operating characteristic (ROC) curves were generated to assess the diagnostic potential of the candidate biomarkers.

The tumor immune microenvironment (TIM) comprises tumor cells, immune cells, fibroblasts, signaling molecules, and the extracellular matrix, and these components critically influence tumor progression. For the immunoinfiltration analysis, gastric cancer samples were analyzed in the cancer genome atlas (TCGA) database (https://tcga-data.nci.nih.gov/tcga/), including the StromalScore, ImmuneScore, and EATIMATE scores. Furthermore, to analyze the abundance of immune infiltrating cells, we used the EPIC and Quantiseq methods. The EPIC method uses gene expression data to analyze infiltration ratios of seven immune cell types, including B cells, tumor-associated fibroblasts (CAFs), CD4^+^ T cells, CD8^+^ T cells, endothelial cells, macrophages, and NK cells. The Quantiseq method employs RNA-seq data to quantify tumor immune status and analyze the proportions of ten different immune cell types.

### 2.9 Downstream and upstream mechanism of the biomarker

Genes typically function within regulatory networks alongside upstream and downstream molecules rather than in isolation. For the downstream mechanism, we used the GeneMANIA database (https://genemania.org/) to identify proteins that might interact with the candidate biomarkers. For the upstream transcription factor mechanism, we used JASPAR (https://jaspar.elixir.no/), the gene transcription regulation database (GTRD) (http://gtrd.biouml.org/), the ChIP-Atlas (https://chip-atlas.org/), and the ChIP-X enrichment analysis 3 (ChEA3) database (https://amp.pharm.mssm.edu/ChEA3) to predict upstream transcription factors of biomarkers. Then calculated the correlation between these transcription factors and the candidate biomarkers.

## 3 Results

### 3.1 Network toxicology prediction of the toxicological effects of 18β-GRA

**FIGURE 1:**
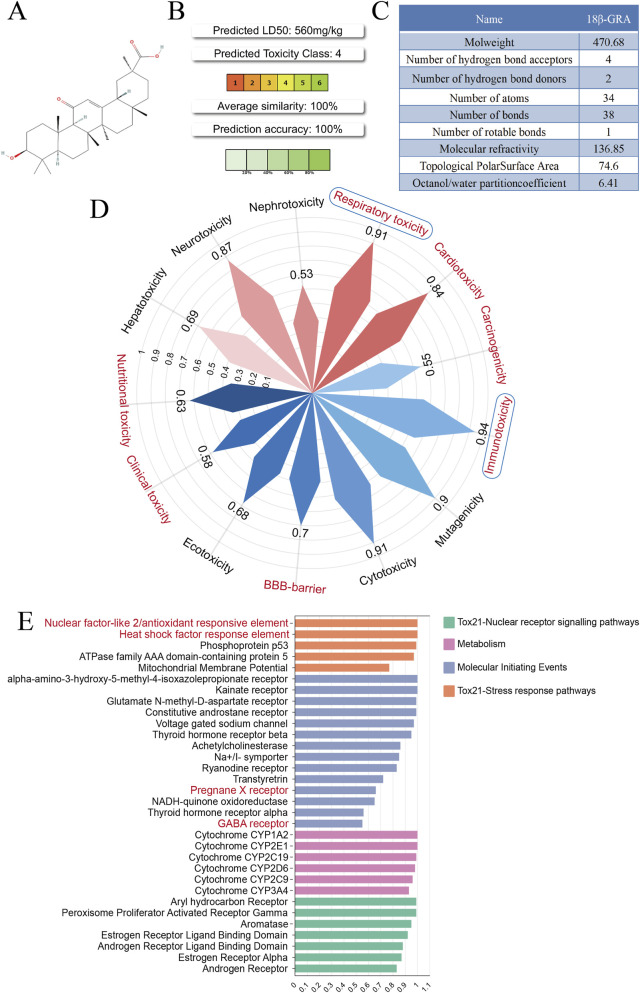
Prediction of the toxicological effects of 18β-GRA based on the ProTox3.0 database. **(A)** The structure of 18β-GRA. **(B)** Oral toxicity of 18β-GRA. **(C)** Basic chemical properties of 18β-GRA. **(D)** Prediction of the toxicological effects of 18β-GRA. The red petals represent organ toxicity, and the blue petals represent toxicological endpoints. The red font represents active 18β-GRA, while the black font represents inactive 18β-GRA. **(E)** Toxicological pathways, molecular initiating events, and metabolic pathways involving 18β-GRA. The red font represents active 18β-GRA, while the black font represents inactive 18β-GRA.

As shown in Figure 1A, the structure of 18β-GRA was retrieved from PubChem. The potential oral toxicity was assessed using the ProTox3.0 database, where higher toxicity grades indicate lower toxicity. Figure 1B shows that 18β-GRA received a grade of 4, suggesting low potential toxicity. Figure 1C shows that 18β-GRA has a molecular weight of 470.68, with four hydrogen bond donors and 2 acceptors. As shown in Figure 1D, 18β-GRA exhibited activity in respiratory toxicity (p = 0.91) and cardiotoxicity (p = 0.84). In addition, potential activity was observed for the following toxicological endpoints: cytotoxicity (p = 0.91), blood-brain barrier (BBB) permeability (p = 0.70), clinical toxicity (p = 0.58), and nutritional toxicity (p = 0.63). Furthermore, we analyzed the effects of 18β-GRA on toxicological pathways, molecular initiating events, and metabolic pathways. As shown in Figure 1E, the compound showed activity in the nuclear factor E2-related factor 2 (Nrf2)/antioxidant responsive element (ARE) and heat shock factor response element pathways, and may bind to GABA receptors. These results indicate that oral administration of 18β-GRA may cause respiratory toxicity, cardiac toxicity and immune toxicity, providing a direction for the subsequent preclinical safety evaluation.

### 3.2 Identification of potential targets for gastric cancer

**FIGURE 2:**
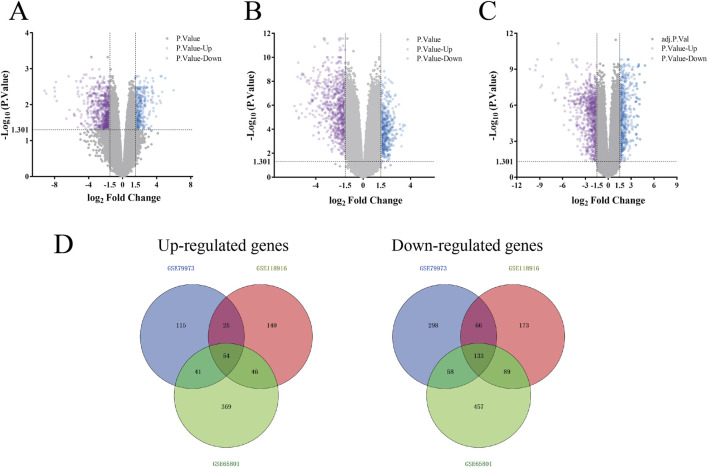
Screening targets related to gastric cancer. **(A–C)** Volcano map of gastric cancer DEGs in GSE79973, GSE118916, and GSE65801 datasets in the GEO database, respectively. Purple represents downregulated genes and blue represents upregulated genes. **(D)** Upregulated and downregulated genes at the intersection of GSE79973, GSE118916, and GSE65801 datasets in the GEO database.

A total of 1,456,530 records were retrieved from the GEO database, and datasets numbered GSE79973, GSE118916, and GSE65801 were finally selected for analysis according to the screening conditions. The GSE79973, GSE118916, and GSE65801 datasets contained 54,675, 49,395, and 42,545 targets, respectively, showing upregulated and downregulated genes with volcanic maps. As shown in Figures 2A–C, the GSE79973 dataset contained 235 upregulated and 555 downregulated DEGs. In the GSE118916 dataset, there were 265 upregulated and 461 downregulated genes. In total, 510 targets were upregulated and 737 targets downregulated in the GSE65801 dataset. As shown in Figure 2D, the targets of the three datasets were overlapped with 54 and 133 upregulated and downregulated targets, respectively.

**FIGURE 3:**
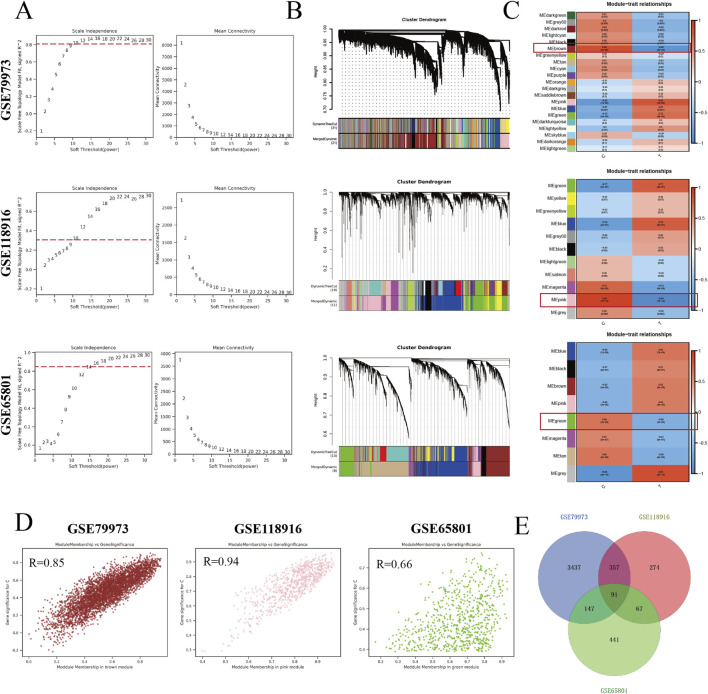
Construct WGCNA to screen gastric cancer-related targets. **(A)** The soft threshold and scale-free topological fit index of the GSE79973, GSE118916, and GSE65801 datasets in the GEO database were determined. **(B)** Module-trait heatmap of the correlation between the clustering gene module and gastric cancer in GSE79973, GSE118916, and GSE65801 datasets. Each module includes the corresponding correlation coefficient and *p-value*. **(C)** Dendrogram of genes clustered by the TOM. **(D)** Scatter plots showing the strongest correlation between specific traits and modular genes. **(E)** Get the genes shared within the brown, pink, and green modules.

All genes in the GSE79973, GSE118916 and GSE65801 datasets were used to construct the WGCNA network. The network correlation coefficient and average connectivity were chosen between 1 and 30 to satisfy the near scale-free distribution of the network while ensuring gene connectivity. In addition, genes with similar expression patterns were assigned to the same module to obtain multiple modules of different colors. As shown in Figures 3A,B, the power selected for the GSE79973 dataset was 10, resulting in a total of 21 modules, the GSE118916 dataset consisted of 11 modules with a power selection of 30, and the GSE65801 had a power selection of 14 for a total of eight modules. As shown in Figures 3C,D, the brown module of GSE79973 (Cor = 0.85, *p-value = 2e-06*), the pink module of GSE118916 (Cor = 0.94, *p-value = 1e-14*) and the green module of GSE65801 (Cor = 0.66, *p-value = 3e-09*) showed the most significant correlations, with 4,032, 789 and 746 genes in the three modules, respectively. As shown in Figure 3E, we selected 91 genes shared by these three modules as potential targets with the highest association with gastric cancer.

### 3.3 Functional enrichment analysis of the target

**FIGURE 4:**
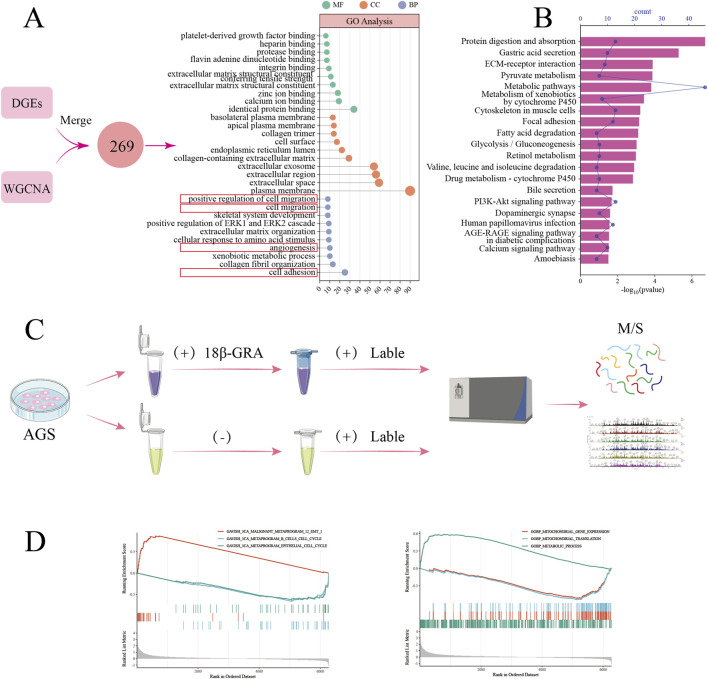
Functional enrichment analysis of gastric cancer-related targets. **(A)** GO and **(B)** KEGG analysis. **(C)** Flow chart of TMT proteomics experiment for 18β-GRA treatment of gastric cancer cells. **(D)** GSEA analysis of genes identified by TMT proteomics.

We identified 187 DEGs from GEO analysis and 91 targets from WGCNA method, yielding 269 common targets for biological pathway exploration. Figure 4A shows GO enrichment results (104 BP, 33 CC, 44 MF terms). The 269 targets were associated with cell migration, adhesion and angiogenesis. Most targets localized to cell membranes, extracellular matrix, and endoplasmic reticulum, functioning in extracellular matrix structural constituents, zinc/calcium ion binding, and protein homo-oligomerization. KEGG analysis revealed 32 pathways, with the top 20 shown in Figure 4B, including protein digestion and absorption, gastric acid secretion, and drug and pyruvate metabolism. TMT-based proteomics was performed in 18β-GRA treated AGS cells in Figure 4C. To ensure comprehensive functional profiling beyond DEG limitations, we performed GSEA on all TMT-quantified proteins. As shown in Figure 4D, the targets were mainly involved in malignant epithelial-mesenchymal transition (EMT) metaprogram, epithelial cell cycle metaprogram, and mitochondrial gene expression, which was consistent with the enrichment results of DEGs.

### 3.4 Machine learning algorithms identify 18β-GRA targets against gastric cancer

**FIGURE 5:**
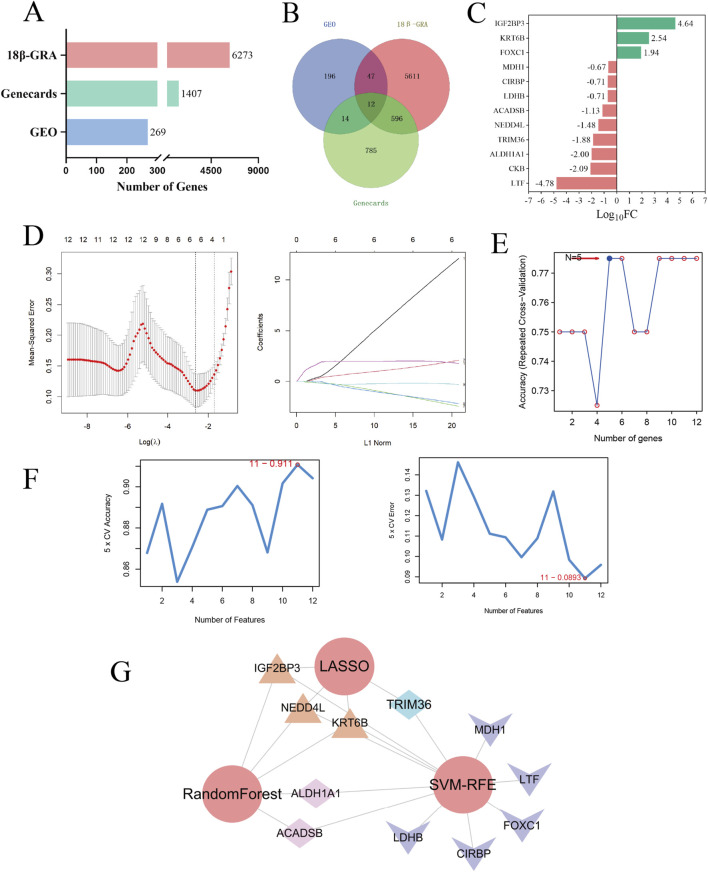
Machine learning algorithm identifies the biomarkers for 18β-GRA in the treatment of gastric cancer. **(A)** The number of targets identified from TMT proteomics, GEO, and GeneCards databases, respectively. **(B)** Overlapping targets of 18β-GRA and gastric cancer. **(C)** Bar graph of LogFC values for overlapping targets. **(D)** Four genes were obtained by the LASSO method. **(E)** The SVMs algorithm extracted five genes. **(F)** The random forest algorithm selected eleven genes. **(G)** Candidate biomarkers were identified by the intersecting LASSO, SVMs and random forest method.

As shown in Figures 5A,B, there were 6,273, 1,407, and 269 potential targets in the TMT proteomics, GeneCards, and GEO databases, respectively, with a total of 12 common targets among the three databases. As shown in Figure 5C, the LogFC values of lactotransferrin (LTF) and insulin like growth factor 2 MRNA binding protein 3 (IGF2BP3) with the most significant expression changes were −4.78 and 4.64, respectively. Figure 5D shows that the LASSO method identified four key targets: IGF2BP3, E3 ubiquitin-protein ligase NEDD4-like (NEDD4L), keratin 6B (KRT6B), and tripartite motif-containing protein 36 (TRIM36). Figure 5E shows that the SVMs algorithm identified eleven top targets: IGF2BP3, NEDD4L, KRT6B, TRIM36, aldehyde dehydrogenase 1A1 (ALDH1A1) and others. Figure 5F shows that the random forest algorithm selected five genes: IGF2BP3, NEDD4L, KRT6B, ALDH1A1, and acyl-CoA dehydrogenase short/Branched chain (ACADSB). As shown in Figure 5G, NEDD4L, IGF2BP3, and KRT6B were obtained by the three learning algorithms and are considered potential biomarkers for 18β-GRA in gastric cancer treatment.

### 3.5 Analyze the clinical relevance of candidate biomarkers

**FIGURE 6:**
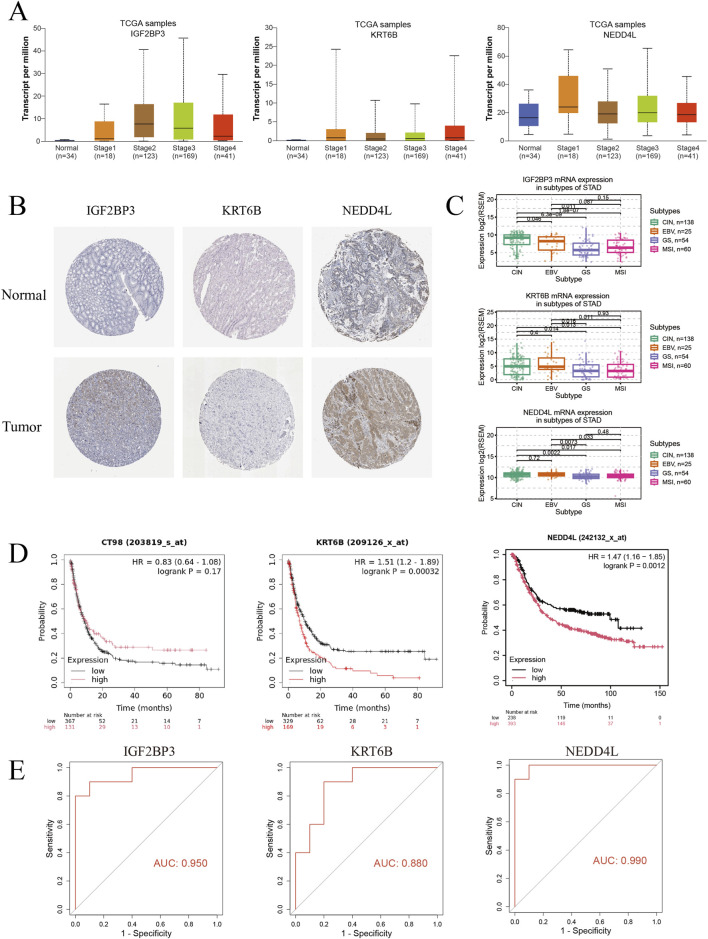
Clinical correlation analysis of the candidate biomarkers. **(A)** The expression levels of the biomarkers at different stages. **(B)** Biomarker immunohistochemical expression was analyzed using the HPA database. **(C)** Expression levels of biomarkers in the four molecular subtypes of gastric cancer. **(D)** Relationship between biomarkers and gastric cancer patient survival prognosis. **(E)** ROC curves of the biomarkers.

As shown in Figure 6A, the expression of IGF2BP3 increased with the progression of gastric cancer, while the expression of NEDD4L increased only in the early stage. This suggests that NEDD4L may be significant for the early clinical diagnosis of gastric cancer. The immunohistochemical results in Figure 6B showed that the expression of brown-yellow particles of NEDD4L increased in gastric cancer tissues, which was consistent with the clinical staging results. The molecular subtypes were associated with gastric cancer patient prognosis, with EBV-positive patients having the best prognosis, followed by microsatellite-stable (MSI) and chromosomally unstable (CIN) patients. Genome-stable (GS) patients had the worst prognosis. As shown in Figure 6C, except for the lack of a significant difference in expression between the CIN and MSI subtypes, the EBV and GS subtypes exhibited different characteristics for IGF2BP3, NEDD4L, and KRT6B, which is significant for selecting targeted therapies. As shown in Figure 6D, overall survival (OS) was significantly shorter in patients with high expression of KTR6B and NEDD4L. Figure 6E shows that the area under the curve (AUC) values for the targets IGF2BP3, NEDD4L, and KRT6B were all greater than 0.8, and NEDD4L had the highest value at 0.99.

**FIGURE 7:**
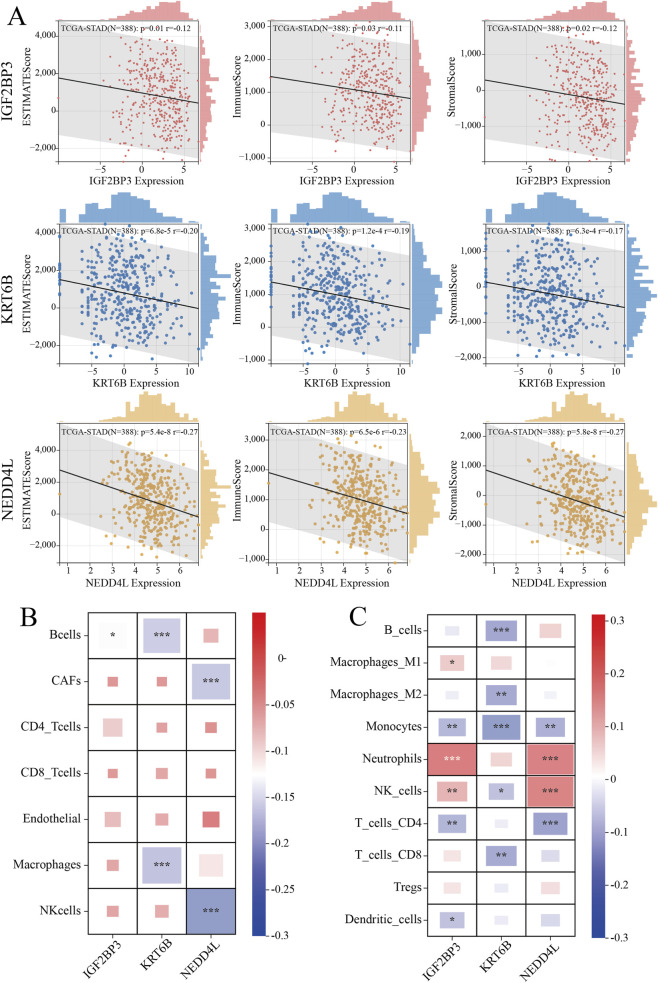
Correlation between candidate biomarkers and immune infiltration. **(A)** Correlation between biomarkers and Stromal, immune, and estimate scores. **(B)** EPIC and **(C)** Quantiseq methods for evaluating immune cell infiltration abundance in biomarkers.

Stromal, immune, and ESTIMATE scores were employed to assess non-tumor cell infiltration in the tumor microenvironment, quantifying stromal content, immune cell infiltration, and tumor purity, respectively. As shown in Figure 7A, IGF2BP3, KRT6B, and NEDD2L demonstrated significant inverse correlations with all three scores, with NEDD4L showing the strongest association. Elevated NEDD4L expression correlated with reduced immune cell proportions, suggesting compromised immune surveillance that may facilitate tumor progression. As shown in Figures 7B,C, complementary EPIC and Quantiseq analyses confirmed immune negative correlations: IGF2BP3, KRT6B and NEDD4L showed broad anti-correlations with multiple immune subsets, which is consistent with the previous results.

### 3.6 Predict the upstream and downstream mechanisms of 18β-GRA against gastric cancer

**FIGURE 8:**
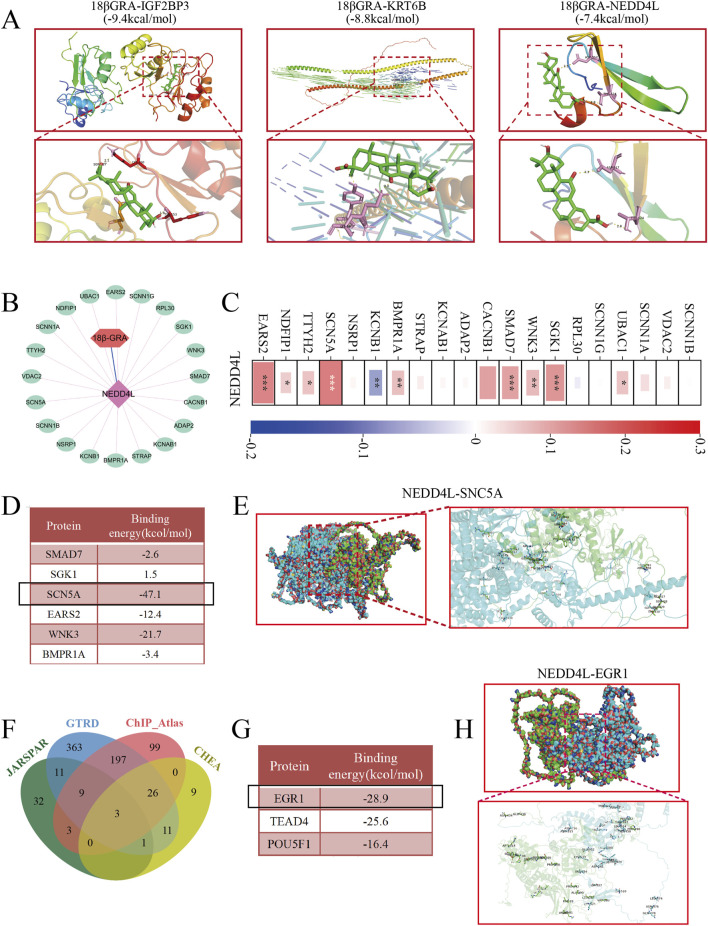
Prediction of the molecular mechanism of 18β-GRA in anti-gastric cancer. **(A)** Molecular docking of 18β-GRA with three candidate biomarkers. **(B)** The GeneMANIA database was used to predict the interacting proteins downstream of NEDD4L. **(C)** Correlation between NEDD4L and downstream proteins. **(D)** Calculate the binding energies of the top six proteins with the highest correlation to NEDD4L. **(E)** Visualization of the protein with the lowest binding energy to NEDD4L. **(F)** Prediction of potential transcription factors upstream of NEDD4L using the JASPAR, GTRD, ChIPAtlas and ChEA3 databases. **(G)** Calculate the binding energies of three transcription factors with NEDD4L. **(H)** Visualize the protein with the lowest binding energy to NEDD4L.

Molecular docking was performed to explore the binding of 18β-GRA to three candidate biomarkers. As shown in Figure 8A, the binding energies of 18β-GRA to IGF2BP3, KRT6B, and NEDD4L were −9.4, −8.8, and −7.4 kcal/mol, respectively. Based on clinical relevance analysis, NEDD4L exhibited the highest ROC curve score (AUC = 0.990). Therefore, we propose that NEDD4L may be a direct target of 18β-GRA. To further explore the potential mechanisms involving NEDD4L, we investigated its upstream and downstream interactors. As shown in Figure 8B, bioinformatic analysis predicted a total of 20 potential downstream interacting proteins. Figure 8C reveals that NEDD4L exhibited a positive correlation trend with all predicted targets except KCNB1. Next, we performed molecular docking to model the interaction between NEDD4L and the top five targets, which were ranked by correlation strength. Figures 8D,E show that NEDD4L has the strongest binding affinity with sodium channel protein type 5 subunit alpha (SCN5A), with a binding energy of −47.1 kcal/mol. To investigate the upstream regulatory mechanisms of NEDD4L, we retrieved its 2,000-base-pair promoter sequence from the NCBI database. We predicted potential transcription factor binding sites in this promoter using the JASPAR, GTRD, ChIPAtlas, and ChEA3 databases. As shown in Figure 8F, three transcription factors were identified in four databases: early growth response 1 (EGR1), TEA domain transcription factor 4 (TEAD4), and POU class 5 homeobox 1 (POU5F1). Then we employed molecular docking to calculate the binding energy between NEDD4L and these candidate transcription factors. As shown in Figures 8G,H, NEDD4L exhibits the strongest binding affinity with EGR1, with a lowest binding energy of −28.9 kcal/mol. In summary, these findings suggest that 18β-GRA may regulate the progression of gastric cancer through the NEDD4L/SCN5A axis. Furthermore, EGFR potentially binds to the NEDD4L promoter and regulates its transcriptional activity.

### 3.7 MD simulation

**FIGURE 9:**
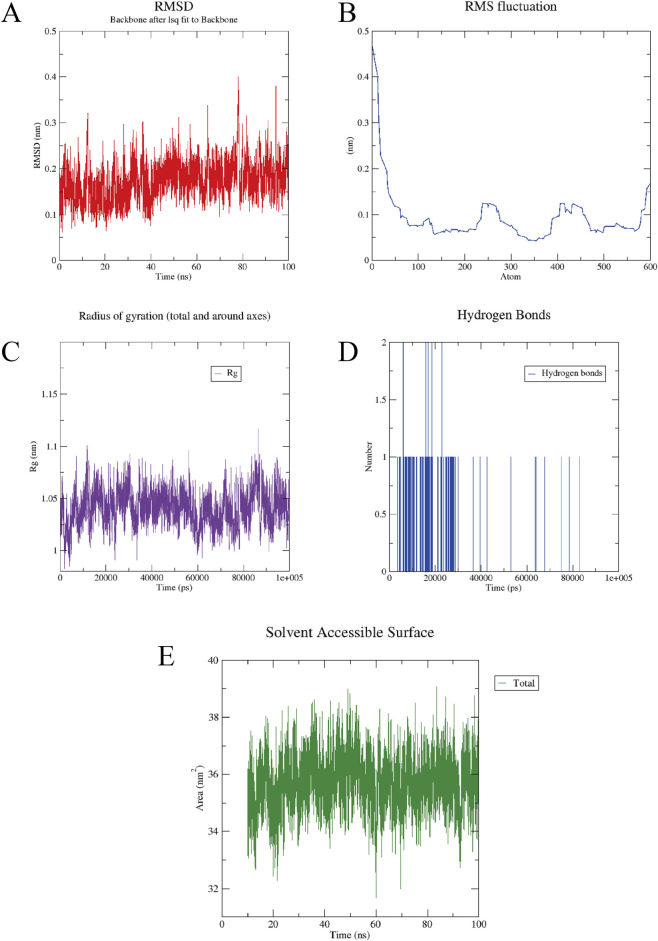
Molecular dynamics simulation results. **(A)** The RMSD of NEDD4L protein. **(B)** The RMSF associated with the number of residue atoms. **(C)** The Rg fluctuation of the protein. **(D)** The number of hydrogen bonds between the 18β-GRA and NEDD4L. **(E)** The SASA analysis of the protein.

To further characterize the atomic-level dynamics of the 18β-GRA-NEDD4L complex, a 100-ns molecular dynamics simulation was performed. RMSD analysis assesses the overall structural stability of the simulated system, with lower values indicating greater stability. Figure 9A shows that the backbone RMSD of the NEDD4L reached 0.174 nm, indicating structural equilibrium was attained. RMSF measures the flexibility of individual protein residues. The average C-α RMSF for NEDD4L was 0.096 nm (Figure 9B), suggesting good residue-level stability. As shown in Figure 9C, the Rg stabilized at 1.041 nm, indicating a compact conformation throughout the simulation. Analysis of hydrogen bonding (Figure 9D) revealed the consistent formation of at least one hydrogen bond between 18β-GRA and NEDD4L. The SASA was calculated to be 35.710 nm^2^ (Figure 9E). In conclusion, these multifaceted analyses demonstrate that 18β-GRA and NEDD4L form a relatively stable complex.

**FIGURE 10:**
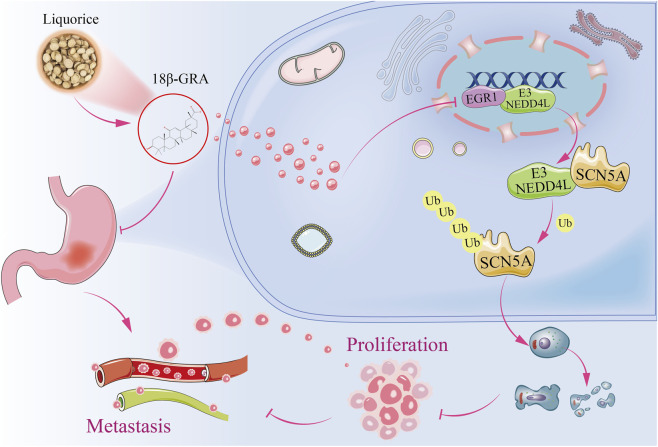
Mechanism of action of 18β-GRA against gastric cancer.

Based on the above results, we proposed a mechanism model to illustrate the potential mechanism of 18β-GRA in gastric cancer, as shown in Figure 10.

## 4 Discussion

Natural plant products are an essential source for developing new anti-cancer drugs. Notably, approximately one-third of Food and Drug Administration (FDA)-approved anti-cancer drugs are currently derived from natural plants (Ashrafizadeh et al., 2020; Ashrafizadeh et al., 2021; Sultani et al., 2021). Glycyrrhetinic acid, a natural pentacyclic triterpenoid, is primarily isolated from the roots of Glycyrrhiza species. This compound exists in two primary isomeric forms: 18α-GRA and 18β-GRA. 18α-GRA exhibits hepatoprotective effects and undergoes irreversible metabolism in the liver by β-D-glucuronidase to form 18β-GRA (Ploeger et al., 2001; Takeda et al., 1996). The anti-cancer activity of 18β-GRA has been extensively studied. Yanfen Cheng demonstrated that 18β-GRA impedes breast cancer progression by inhibiting macrophage M2 polarization through activation of the c-Jun N-terminal kinase (JNK) signaling pathway (Cheng et al., 2023). Furthermore, recent research indicates that 18β-GRA exerts anti-small cell lung cancer effects by promoting peroxiredoxin-6 (Prdx6) and caspase-3-mediated mitochondrial apoptosis (Guo et al., 2024). Consistent with prior studies, our research confirmed the significant anti-cancer activity of 18β-GRA through the induction of autophagy, cell cycle arrest, and apoptosis. The development of natural product-derived anti-cancer agents represents a significant research focus, as demonstrated by 18β-GRA. Its scaffold has three modifiable groups that serve as sites for derivative synthesis: C-3 hydroxyl, C-11 carbonyl, and C-30 carboxylic acid (Roohbakhsh et al., 2016). Over 400 derivatives have been developed that exhibit potent cytotoxicity against multiple carcinomas, including breast, cervical, ovarian, lung, and thyroid cancers, at concentrations of 1.7–8.5 μM. Notably, some derivatives are more effective than traditional drugs such as doxorubicin (IC_50_ = 2.4 μM) and gefitinib (IC_50_ = 6.7 μM) in efficacy against cancer (Gao et al., 2010; Zheng et al., 2020). In conclusion, 18β-GRA is a promising lead compound for developing future chemotherapeutic agents.

Nevertheless, comprehensive toxicity assessment remains essential in drug development. Computer toxicity models integrate toxicology, biostatistics, systems biology, and computer science to predict the potential toxicological effects of compounds. These models have been verified through cross-validation and external validation sets. The ProTox3.0 database suggested potential respiratory and cardiac risks associated with 18β-GRA. Paradoxically, evidence suggests that 18β-GRA improves oxygen-induced bronchopulmonary dysplasia in premature infants and has potential as an anti-respiratory syncytial virus agent (Qing et al., 2022; Özkan et al., 2023). Although toxicological endpoints suggest potential impairment of the immune system, Xuan Ma reported increased CD8^+^ T-cell activity and hepatoprotective effects (Ma et al., 2024; L et al., 2013). Studies have shown that excessive licorice consumption can lead to hyperaldosteronism and arrhythmia (Yoshino et al., 2021; Omar et al., 2012). However, discontinuing consumption and taking electrolyte supplements can alleviate these symptoms (Attou et al., 2020). Our previous experiments revealed that 18β-GRA could maintain a survival rate above 90% for normal gastric mucosal cells at doses below 100 μmol/L. However, this concentration was sufficient to significantly inhibited the growth of gastric cancer cells (Li et al., 2023). In summary, maintaining within the recommended dosage range of 18β-GRA may reduce potential toxicity, and administering fluid replacement therapy after discontinuation can mitigate complications.

With the rapid development of computer science, high-throughput sequencing technology and machine-learning algorithms are helping researchers to further explore the disease. We used machine learning algorithms, including WGCNA, LASSO, SVMs, and random forest methods, to identify potential biomarkers of 18β-GRA for gastric cancer. After combining the above methods, we ultimately identified three candidate biomarkers: NEDD4L, IGF2BP3, and KRT6B. Using molecular docking technology, we simulated the binding of these candidate markers to 18β-GRA. All three proteins exhibited binding energies below < −7.0 kcal/mol, indicating a relatively high likelihood of binding *in vivo*.

Ubiquitination is a type of post-translational protein modification mediated by three types of enzymes: ubiquitin-activating enzymes (E1s), ubiquitin-conjugating enzymes (E2s), and ubiquitin ligases (E3s). Ubiquitin ligases play a key role in determining the specificity of target protein binding (d'Azzo et al., 2005). Over 600 E3 ligases have been identified, with the E6AP C-Terminus Homologous (HECT) family representing a major class (Jayaprakash et al., 2022; Wang et al., 2020a). HECT ligases are classified by their N-terminal domain and include the NEDD4, HRC, and HECT subfamilies. NEDD4L, a well-studied member of the NEDD4 subfamily, has multiple protein-binding domains, including the C2 domain and cysteine residues (Xu et al., 2022; Jiang et al., 2019). These domains contribute to NEDD4L’s broad functional diversity. NEDD4L binds to and promotes the degradation of downstream target proteins via lysosomes or proteasomes. Studies have shown that NEDD4L inhibits esophageal squamous cell carcinoma by inducing MYC ubiquitination and decreasing SLC1A5 expression (Cheng W. et al., 2022). In colorectal cancer, NEDD4L suppresses tumorigenesis by promoting the ubiquitin-mediated degradation of phosphatase and tensin homolog (PTEN) and forkhead box A1 (FOXA1) (Kim et al., 2008; Yue et al., 2021). Research has confirmed that NEDD4L expression is increased in gastric cancer tissues and associated with a poor prognosis. These results are consistent with our bioinformatics findings (Sun et al., 2014; Wang et al., 2010). The clinical relevance analysis revealed NEDD4L’s prominent role, characterized by increased expression in early gastric cancer and EBV subtypes. Furthermore, treatment with 18β-GRA significantly decreased NEDD4L expression in gastric cancer cells, suggesting its potential as a diagnostic and therapeutic biomarker for the disease.

Gene regulation occurs through multilevel interactions within biological networks. The N-terminal domains of NEDD4L facilitate these interactions. Using public databases, we predicted NEDD4L-interacting proteins and simulated binding affinity via molecular docking. Notably, NEDD4L exhibited the strongest binding energy of −47.1 kcal/mol with SCN5A, suggesting the formation of a stable complex. The SCN5A gene encodes the Nav1.5 voltage-gated sodium channel α-subunit, which is essential for initiating and propagating action potentials (Catterall et al., 2020). Studies indicate that the C-terminal PY-motif of SCN5A binds NEDD4L’s WW domains, triggering SCN5A ubiquitination and contributing to fatal arrhythmias (Wang et al., 2020b). Consistent with our bioinformatic predictions, SCN5A expression increased in breast and colon cancers (Leslie et al., 2024; Sui et al., 2021). Therefore, we hypothesize that 18β-GRA promotes SCN5A ubiquitination via NEDD4L to inhibit gastric cancer progression. EGR1 plays an important role in cancer by promoting tumorigenesis through enhanced cyclin D1 expression in prostate cancer and by interacting with vascular endothelial growth factor A (VEGFA) to stimulate angiogenesis in lung cancer (Xiao et al., 2005; Shimoyamada et al., 2010). However, EGR1 can also suppress tumorigenesis by activating p53 (Yu et al., 2007). Bioinformatics analyses revealed decreased EGR1 expression alongside increased NEDD4L expression in gastric cancer, suggesting EGR1 may bind the NEDD4L promoter to regulate its transcription.

## 5 Conclusion

While our findings reveal a potential link between 18β-GRA and gastric cancer, reliance on public databases for predictions may limit causal interpretation. Further verification of the regulatory relationship between 18β-GRA and NEDD4L/SCN5A is necessary in cell experiments. Additionally, clinical sample data are essential to establish the therapeutic relevance of 18β-GRA in delaying disease progression.

## Data Availability

The TMT proteomics data of this article are available on reasonable request from the corresponding author. The remaining data analyzed in this study are publicly available: The toxicological data of 18β glycyrrhetinic acid was obtained from the ProTox3.0 database (https://tox.charite.de/protox3/), while the gastric cancer tissue chip data were obtained from the GEO database (https://www.ncbi.nlm.nih.gov/geo/).
